# Quantitative assessment of common genetic variations in HLA-DP with hepatitis B virus infection, clearance and hepatocellular carcinoma development

**DOI:** 10.1038/srep14933

**Published:** 2015-10-14

**Authors:** Lei Yu, Yi-ju Cheng, Ming-liang Cheng, Yu-mei Yao, Quan Zhang, Xue-ke Zhao, Hua-juan Liu, Ya-xin Hu, Mao Mu, Bi Wang, Guo-zhen Yang, Li-li Zhu, Shuai Zhang

**Affiliations:** 1 The Affiliated Hospital, Guizhou Medical University, Beijing Road 9, Guiyang 550004, Guizhou, China; 2 The First Affiliated Hospital, Soochow University, Shizi Street 188, Suzhou 215006, Jiangsu, China; 3 Department of Eugenics and Genetics, Guiyang Maternal and Child Health-Care Hospital, Ruijin South Road 63, Guiyang 550003, Guizhou, China

## Abstract

Hepatitis B virus (HBV) infection is the predominant risk factor for chronic hepatitis B (CHB), liver cirrhosis (LC) and hepatocellular carcinoma (HCC). Recently, genome-wide association studies have identified human leukocyte antigen (HLA)-DP polymorphisms (rs3077 and rs9277535) as a new chronic HBV infection susceptibility locus. Since then, the relationship between HLA-DP polymorphisms and various outcomes of HBV infection has been reported. However, the results have been inconclusive. To derive a more precise estimation of the relationship between HLA-DP polymorphisms and various outcomes of HBV infection, a meta-analysis of 62,050 subjects from 29 case-control studies was performed. We found that rs3077 and rs9277535 in HLA-DP significantly decreased HBV infection risks and increased HBV clearance possibility in a dose-dependent manner. In the subgroup analysis by ethnicity, study design and sample size, significant associations were found for these polymorphisms in almost all comparisons. Meanwhile, haplotype analyses of the two polymorphisms revealed a significant association between the combination of these alleles and HBV infection outcomes. However, no significant results were observed in HCC development. Our results further confirm that genetic variants in the HLA-DP locus are strongly associated with reduced HBV infection and increased the likelihood of spontaneous viral clearance.

Hepatitis B virus (HBV) infection is a major global health concern, with more than 2 billion people infected, of whom 400 million are chronic carriers^1^,^2^. Although some HBV carriers spontaneously eliminate the virus, carriers are at an increased risk of developing liver cirrhosis (LC), hepatic decompensation, and hepatocellular carcinoma (HCC) development^3^, especially in the endemic areas of Southeast Asia, China, Japan, and Sub-Saharan Africa^4^. In addition to the viral and environmental factors^5^, results from twin studies, family clustering studies and studies of differences between ancestry groups have suggested that host factors are critical in determining the outcome of HBV infection^6^.

Over the past decade, many efforts have been put into the deciphering the genetic architecture for HBV persistence. From a long list of candidate genes, may variants have been inconsistently associated with HBV infection: *IFNG*^7^, *TNF*^7^, *ESR1*^8^, *VDR*^9^, *MBP*^10^, *CTLA4*^11^, and the human leukocyte antigen (HLA) class II^12^. However, because the exact mechanisms of HBV persistence are yet to be elucidated completely, the candidate-gene approach is limited in power to detect novel disease-susceptibility genes. Recently, a genome-wide association study (GWAS) identified two single-nucleotide polymorphism (SNPs) in HLA-DP (rs3077 and rs9277535) for persistent HBV infection in Japanese and Thai populations^13^. Replication of initial GWAS findings is considered a gold standard for reporting genotype–phenotype associations. As stated by McClellan and King, many if not most of the genetic polymorphisms that are reported to be associated with common disorders in GWA studies are factually spurious associations caused by subtle differences in ancestry between the populations being studied (known as “cryptic population stratification”)^14^. To date, many case–control studies have been carried out to investigate the role of the two SNPs in HLA-DP in relation to outcomes of HBV infection among various populations. Genetic association studies can be problematic to reproduce due to insufficient power, ethnic diversity, multiple hypothesis testing, population stratification, phenotypic heterogeneity and publication bias. Therefore, we carried out a comprehensive meta-analysis on all eligible studies to estimate relationship between HLA-DP polymorphisms (rs3077 and rs9277535) and HBV infection outcomes as well as to quantify the between-study heterogeneity and potential bias.

## Results

### Characteristics of the included studies

Based on our search strategy, the primary screening produced 369 potentially relevant articles. Study selection process was shown in Figure S1. Overall, 28 published studies and one unpublished data involving 29,790 HBV carriers, 7,804 subjects with HBV natural clearance, 4,864 HCC patients, and 24,456 healthy individuals were finally included^13^,^15^,^16^,^17^,^18^,^19^,^20^,^21^,^22^,^23^,^24^,^25^,^26^,^27^,^28^,^29^,^30^,^31^,^32^,^33^,^34^,^35^,^36^,^37^,^38^,^39^,^40^,^41^. Of the subjects, most were Asians, only 3.1% were of other ethnic origins. The main study characteristics were summarized in Table S1. There was a wide variation in the risk allele frequency of the two polymorphisms among the controls across different ethnicities (Figure S2).

### Association between HLA-DPA1 rs3077-A and HBV infection, HBV spontaneous clearance, HCC development

**Figure 1:**
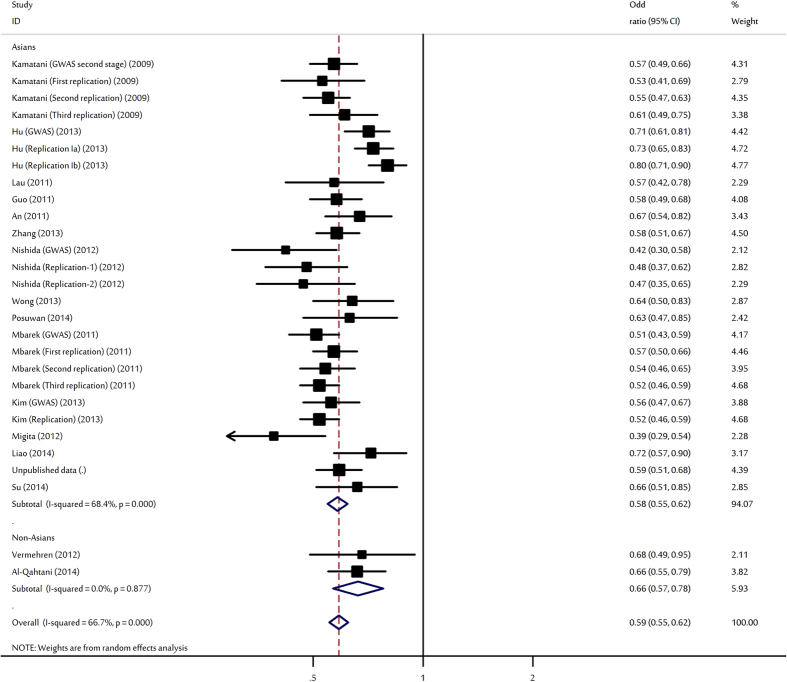
Forest plot for the meta-analysis of the association between rs3077 polymorphism and HBV infection stratified by ethnicity.

The evaluations of the association of HLA-DPA1 rs3077-A polymorphism with various outcomes of HBV infection are shown in Table 1. Overall, the HLA-DPA1 rs3077-A allele was shown to protect against HBV infection with per-allele OR of 0.59 (95% CI: 0.55–0.62, P < 10^−5^; Fig. 1). Significant associations were also found for AG heterozygote (OR = 0.55, 95% CI: 0.51–0.61, P < 10^−5^) and AA homozygote (OR = 0.39, 95% CI: 0.35–0.45, P < 10^−5^). In the subgroup analysis by ethnicity, study design and sample size, significantly decreased HBV infection risks were found for the polymorphism in almost all comparisons.

**Figure 2:**
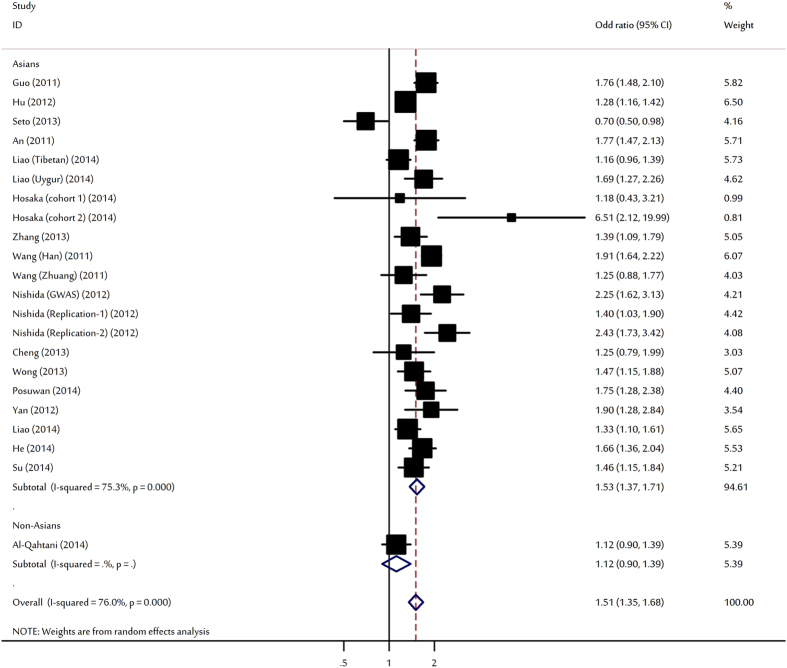
Forest plot for the meta-analysis of the association between rs3077 polymorphism and HBV clearance stratified by ethnicity.

For HBV clearance, our meta-analysis shown that individuals carrying the HLA-DPA1 rs3077-A allele had a significantly higher chance of spontaneous clearance upon HBV infection (OR = 1.51, 95% CI: 1.35–1.68, P < 10^−5^; Fig. 2). Significant increased HBV natural clearances were also observed for heterozygote and homozygote. When stratifying for ethnicity, significant associations were only found among Asians. Subsidiary analyses according to study design and sample size, significant associations were also maintained for all comparisons (Table 1).

Compared with active symptomatic HBV-carriers (e.g., LC, CHB), there is no significant difference in rs3077 genotype distribution in asymptomatic HBV-carriers (Table 1).

The data on genotypes of rs3077 among HBV-induced HCC patients and HBV carriers were available in 7 studies. No evidence of any gene-disease association was obtained (Table 1).

### Association between HLA-DPB1 rs9277535-A and HBV infection, HBV spontaneous clearance, HCC development

**Figure 3:**
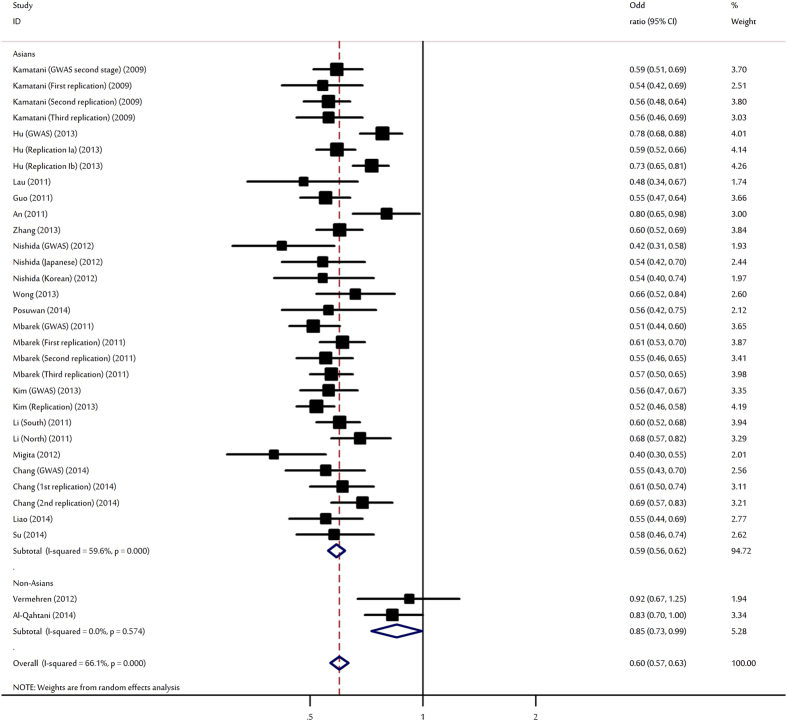
Forest plot for the meta-analysis of the association between rs9277535 polymorphism and HBV infection stratified by ethnicity.

Overall, there was evidence of an association between the decreased risk of HBV infection and the rs9277535-A variant in different genetic models when all the eligible studies were pooled into the meta-analysis (Table 2). Using random-effects model, the summary per-allele OR of the rs9277535-A variant for HBV infection was 0.60 (95% CI: 0.57–0.63, P < 10^−5^; Fig. 3). Significantly decreased HBV infection risks were also found for those heterozygote (OR = 0.56, 95% CI: 0.52–0.60, P < 10^−5^) and homozygous for the minor A allele (OR = 0.39, 95% CI: 0.35–0.43, P < 10^−5^) when compared with the wild type genotype. In the stratified analysis by ethnicity, study design and sample size, significant associations were detected in all genetic models for the polymorphism (Table 2).

**Figure 4:**
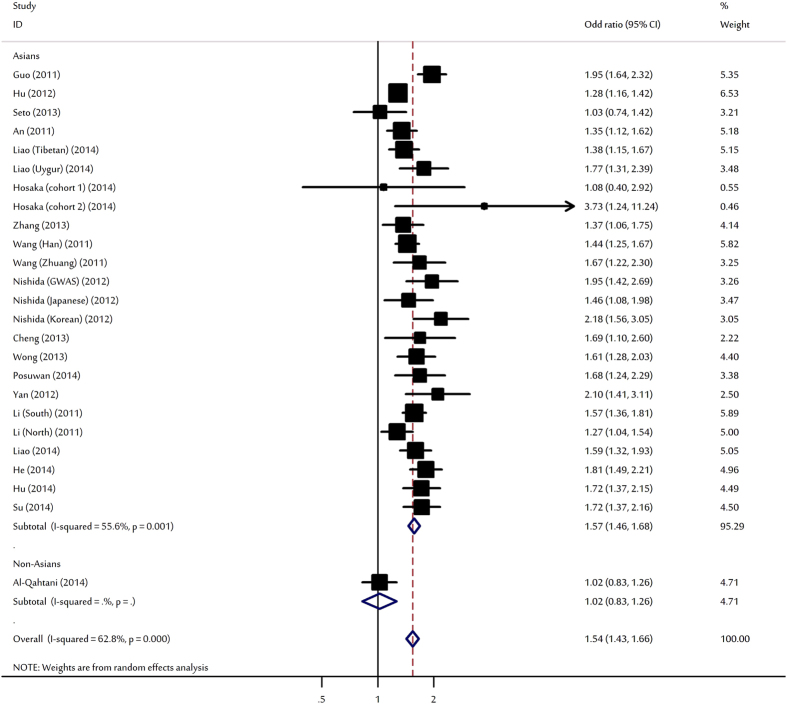
Forest plot for the meta-analysis of the association between rs9277535 polymorphism and HBV clearance stratified by ethnicity.

Meta-analyses showed that rs9277535-A was significantly associated with increased HBV clearance with per-allele OR of 1.54 (95% CI: 1.43–1.66, P < 10^−5^; Fig. 4). Significant associations were found for heterozygote and homozygote. Subgroup analysis for rs9277535 and HBV clearance was also performed to explore the sources of heterogeneity. In the subgroup analyses by ethnicity, the SNP significantly increased natural HBV clearances only among Asians. Subsidiary analyses by study design yielded OR of 1.95 (95% CI: 1.42–2.69) and 1.53 (95% CI: 1.41–1.65) for GWAS and replication studies, respectively. After stratification for sample size, significant results still maintained irrespective to sample size (Table 2).

When HBV infection outcomes as asymptomatic HBsAg carrier (AsC) and HCC development were considered, no significant associations were detected (Table 2).

### Haplotype analysis

Haplotype analyses between rs3077 and rs9277535 polymorphisms were performed in the 11 articles, involving 4,044 HBV carriers, 5,357 HBV natural clearances and 2,730 healthy controls. When compared with the most frequent G-G haplotype, all other haplotypes containing variant alleles of the two SNPs were associated with decreased HBV infection (OR range from 0.57 to 0.82, Table S2). In addition, these haplotypes were also associated with higher chance of HBV natural clearance (OR range from 1.32 to 1.65, Table S3). Results from Haplotype analyses were consistent with the single SNP analysis.

### Credibility of genetic association

To assess the credibility of genetic associations, we considered the BFDP (Table S4–S5) and the Venice criteria (Table S6). Applying these filters indicate that the two variants were graded strong for cumulative evidence of association with HBV infection and HBV clearance. In addition, associations of HBV infection and HBV clearance were maintain statistically significant after Bonferroni correction for multiple genetic models for the 2 SNPs.

### Heterogeneity analyses

In view of significant heterogeneity and to seek for its potential sources, we performed a panel of meta-regression analysis. In meta-regression analysis, sample size, study quality, mean age of cases and controls, sex distribution among cases and controls, ethnicity, study design, did not significantly correlated with the magnitude of the genetic effect for rs3077 and rs9277535 (P > 0.05 for all). Furthermore, Galbraith plot analyses of all included studies were used to assess the potential sources of heterogeneity (Figure S3–S6).

### Association of rs3077-A variant with HLA-DPA1 mRNA expression

To further explored potential function, mRNA expression level of HLA-DPA1 by rs3077 genotypes from peripheral blood mononuclear cells (PBMC) and brain tissues of European descent was obtained from SNPExpress. When pooled all available data together, significantly increased of transcript expression levels by A allele carriers was found for HLA-DPA1 in PBMC (P = 0.017; Figure S7a) and in brain tissues (P = 0.003; Figure S7b).

### Sensitivity analyses and publication bias

A single study involved in the meta-analysis was deleted each time to reflect the influence of the individual dataset to the pooled ORs, and the corresponding pooled ORs were not qualitatively altered for rs3077 (Figure S8–S9) and rs9277535 (Figure S10–S11), suggesting that the results of this meta-analysis are stable. Funnel plot and Egger’s test were performed to access the publication bias of the literatures. The shapes of the funnel plot for the per-allele comparison of the A allele and the G allele seemed symmetrical (Figure S12–S15). The statistical results still did not show publication bias in these studies (Egger’s test: P > 0.05, for all).

## Discussion

The nature history of HBV infection is complicated and identifying biomarkers could facilitate prediction and prevention of vulnerable populations with higher risk to develop CHB and even worse outcomes, such as LC and HCC. Accumulating evidence indicated that host genetic factors play a major role in the persistence of HBV infection^42^. Recent GWAS studies have suggested that certain variations in the HLA-DP regions are associated with protection against chronic hepatitis B as well as viral clearance^13^,^20^,^24^. After that, many replications studies have been conducted to explore the relationship between HLA-DP polymorphisms (rs3077 and rs9277535) and various outcomes of HBV infection. As significant differences in allele frequencies and the prevalence of HBV infection among various populations exist, it is, therefore, important to quantitatively assess the effects of the GWAS-identified markers in different ethnic populations and explore potential heterogeneity of published data. This is the most comprehensive meta-analysis examining the association of rs3077 and rs9277535 polymorphisms on HLA-DP regions and its relationship to outcomes HBV infection. Its strength was based on the accumulation of data giving greater information to detect significant differences. In total, the meta-analysis involved 29 studies including 62,050 subjects.

In this large-scale meta-analysis, the combined evidence confirmed that two SNPs (rs3077 and rs9277535) at HLA-DP locus were significantly associated with decreased HBV infection risk as well as increased spontaneous viral clearance. A panel of subgroup analysis based on ethnicity, sample size and study design were performed and significant associations maintained in almost all comparisons for the two SNPs. However, the heterogeneity of OR is high in our data, especially in the studies for Asian populations. Indeed, the Asian population reports in the subgroup analysis include a mixture of populations from very distant areas. The presence of heterogeneity can result from differences in environmental factors, lifestyle and host-related physical factors^43^. Furthermore, HBV genotype, viral activity, duration of infection may also contribute such heterogeneity^28^,^44^. Applying Venice criteria and the BFDP indicate that associations with the 2 SNPs represent the most credible findings.

When stratified by ethnicity, inconsistent association results for the two SNPs were observed in Asians and non-Asian populations. In fact, differences in genetic backgrounds may attribute to these results. For example, the risk allele distribution of rs3077 varies between Asians, and non-Asians, with a prevalence of 47, and 76%, respectively. Such a result could also be due to the limited number of studies among non-Asians, which had insufficient statistical power to detect a slight effect. On the other hand, different populations usually have different linkage disequilibrium (LD) patterns. A polymorphism may be in close linkage with another nearby causal variant in one ethnic population, but not in another. Furthermore, it is possible that variation at this locus has modest effects on outcomes of HBV infection, but environmental factors may predominate in the progress of HBV infection, and mask the effects of this variation. Specific environmental factors like aflatoxin B1 exposure^2^ and prevalence of HBV^3^ have been already well studied in recent decades.

If genetic susceptibility to HBV infection is, in part, mediated through gene polymorphisms, it is possible that the combinations of certain genotypes may be more discriminating as risk factors for HBV infection than a single locus genotype. Haplotypes analyses of the rs3077-A and rs9277535-A alleles reveal the association between the combination of these alleles in protection against HBV infection as well as beneficial effect of spontaneous viral clearance.

The rs3077 and rs9277535 SNPs are located within 3′-UTR of HLA-DPA1 and HLA-DPB1 gene, respectively. It is possible that they act as the binding site of microRNA and thus affect both the translation and stability of mRNA. SNPs located at miRNA-binding site are likely to disrupt miRNA-target interaction, and result in the deregulation of target gene expression^45^. We therefore compared the mRNA expression levels of HLA-DPA1 by the rs3077 genotypes and found that rs3077-A allele is associated with higher HLA-DPA1 expression. More recently, the A alleles of HLA-DP rs3077 and rs9277535 were reported to be strongly associated with increased levels of mRNA expression of HLA-DPA1 and HLA-DPB1, respectively, in normal liver tissues^46^. Higher levels of HLA-DPA1 on target cell surfaces might be more effective in presenting viral antigen to CD4 + T helper cells, leading to an impaired immune response to viral invasion or to the resolution of HBV infection^47^. Furthermore, rs3077 showed protective effects for response to hepatitis B vaccination^48^. On the other hand, these two SNPs may be in close linkage with another nearby causal variant. Therefore, re-sequencing and fine mapping of this region to identify putative causal variants, combined with functional evaluation, are required.

Chronic HBV infection seems to be the most important risk factor for HCC^4^,^49^. However, no significant associations were observed for asymptomatic HBsAg carrier or HBV-related HCC for rs3077 and rs9277535. One of the possible reasons could be the high complexity of multivariate interactions between the genomic information and the phenotype that is manifesting. HCC development is a multiple process which links to causative factors such as environmental toxins (e.g., Aflatoxin B1), alcohol drinking and smoking habits (two of the main recognized HCC risk factors), lifestyle (e.g., vegetables, fruit consumption), and HBV genotype variations^50^,^51^.

Compared with the previous meta-analysis^52^, the present study is much larger, with more than three times as many subjects as the earlier study. In addition, we assessed not only the effect on HBV infection and viral clearance but also the effects on HBV activity and HCC development. Furthermore, we also investigated whether the haplotypes were associated with HBV infection or clearance. Moreover, we explored potential sources of heterogeneity across studies and the possibility of publication bias.

In interpreting the results, some limitations of this meta-analysis should be addressed. Firstly, the vast majority of subjects in the study are of East Asian descent, and statistical power for analyses in other ethnicities is limited. Because the sample size was relatively smaller for Caucasian studies, the main conclusions from this manuscript are based on analyses among East Asian populations. Further studies including a wider spectrum of ethnic populations are necessary. Secondly, our results were based on unadjusted estimates, while a more precise analysis should be conducted if all individual raw data were available, which would allow for the adjustment by other co-variants including alcohol abuse, aflatoxin B1 exposure, cigarette smoking and other lifestyle. Finally, lacking the original data limited our further evaluation of potential interactions clinical outcomes (e.g., ALT level, AST level, Albumin level) and viral backgrounds (e.g., HBV genotype, viral load).

Despite these limitations, findings of the present study showed that SNPs rs3077 and rs9277535 at HLA-DP locus protected against HBV infection and increased chance of HBV clearance; while the importance of these polymorphisms as a predictor of HCC may be limited.

## Methods

### Identification and screening of relevant studies

The present meta-analysis was performed according to the guideline of PRISMA statement. Genetic association studies published before the end of March 2015 on various outcomes of HBV infection and the two SNPs (rs3077 and rs9277535) at HLA-DP were identified through a search of PubMed, ISI Web of Knowledge, EMBASE, SCOPUS, and Cochrane databases without language restriction. Search term combinations were keywords relating to HBV (e.g., “chronic HBV infection”, “chronic hepatitis B”, “hepatitis B Virus”, “HBV clearance”, “liver cirrhosis”, “hepatocellular carcinoma”) in combination with words related to HLA-DP (e.g., “rs3077”, “rs9277535”, “HLA”, “human leukocyte antigen-DP”). The titles and abstracts of potential articles were screened to determine their relevance, and any clearly irrelevant studies were excluded. The full texts of the remaining articles were read to determine whether they contained information on the topic of interest. Reference lists of included studies and relevant reviews were hand searched for additional eligible studies.

### Criteria for inclusion

The included studies have to meet the following criteria: (1) case–control or cohort studies to evaluate the association between polymorphisms at HLA-DP and various outcomes of HBV infection; (2) original papers containing independent data; (3) Identification of HBV infected cases was confirmed pathologically; (4) available genotype distribution information or odds ratios (ORs) with its 95% confidence intervals (CIs) and P value; (5) genotype distribution of control group must be consistent with Hardy–Weinberg equilibrium (HWE). The major reasons for exclusion of studies were (1) overlapping data, (2) case-only studies, and (3) review articles.

### Quality assessment and data extraction

For association studies with inconsistent results on the same polymorphisms, the methodological quality should be assessed by appropriate criteria to limit the risk of introducing bias into meta-analyses. A procedure known as ‘Newcastle – Ottawa Scale (NOS)’ has been used to assess the quality of association studies. Detailed procedure of the quality assessment was previously described^53^.

Not all researchers use the same HLA-DP SNPs, and most articles reported results for multiple SNPs (uniquely identified by their rs number). We report herein 2 common SNPs (rs3077, rs9277535) that were included in all but 3 articles^27^,^31^,^34^. The remaining 3 articles used 1 additional SNP (rs9277378), as this SNP had a high level of linkage disequilibrium with rs9277535 (D’ = 1.00, R^2^ = 0.954) in the HapMap Han Chinese in Beijing (CHB) and Japanese in Tokyo (JPT) Populations^54^. Data extraction was performed independently by two reviewers. For each study, the following variables were collected according to a fixed protocol: the first author, published year, ethnicity, identification of cases, HBV genotype, viral activity, duration of infection, ALT (alanine aminotransferase) level, AST (aspartate aminotransferase) level, Albumin level, bilirubin level, definitions of control groups, age, sex, study design, source of controls, Hardy–Weinberg equilibrium (HWE) status among controls, number of cases and controls, outcomes of HBV infection (CHB, natural clearance, AsC, LC, HCC), number of genotypes and genotyping methods. For studies including subjects of different ethnic groups, data were extracted separately and categorized. Meanwhile, different case-control groups in one study were considered as independent studies. Review reports from the two were then compared to identify any inconsistency, and differences were resolved by further discussion among all authors.

### Genotype and gene expression correlation analysis

The data on rs3077 genotype and *HLA-DPA1* expression levels were available by SNPExpress tool^55^. The transcript (mRNA) expression data were detected by using genome-wide expression arrays from peripheral blood monocytes of 80 healthy individuals and brain tissues of 93 healthy individuals. Genome-wide genotyping was performed using genechips.

### Statistical analysis

The data from each SNP was divided into three groups: chronic HBV infection vs. healthy controls; spontaneous clearance individuals vs. chronic HBV infection and HCC vs. HBV carriers. The strength of the association between various outcomes of HBV infection and the two polymorphisms (rs3077, rs9277535) was estimated using ORs, with the corresponding 95% CIs. The per-allele OR of the risk allele of these polymorphisms was compared between cases and controls. Then, we estimated the risks of the heterozygote and homozygous genotypes compared with the wild-type homozygote^56^. Cochran’s chi-square-based Q statistic test and I^2^ statistics was performed to evaluate possible heterogeneity between the individual studies. Random effects and fixed effect summary measures were calculated as inverse-variance-weighted average of the log odds ratio^57^,^58^. The results of random effects summary were reported in the text because it takes into account the variation between studies. Sources of heterogeneity were investigated by stratified meta-analyses based on ethnicity (Asians or Non-Asians), study design (GWAS or replication study), and sample size (≥500 cases or, <500 cases). Furthermore, ethnic group, study design, sample size, mean age of cases and controls and sex distribution in cases and controls were analysed as covariates in meta-regression. Sensitivity analysis was performed by removing each individual study in turn from the total and re-analysing the remainder. Publication bias was assessed with the funnel plot and Egger test. All P values are two-sided at the P = 0.05 level. Statistical analyses were carried out using the STATA software version 10.0 (Stata Corporation, College Station, TX, USA) and SAS (version 9.1; SAS Institute, Cary, NC, USA).

### Credibility of genetic association

For statistically significant associations identified by meta-analyses, Venice criteria and BFDP were applied to assess the credibility of the evidence^59^. Venice criteria details are published elsewhere^60^. The BFDP threshold for noteworthiness was set up to be equal to 0.20, based on the assumption that a false discovery would be four times more costly than a false non-discovery. We chose to calculate BFDP values for two levels of prior probabilities: at a medium or low prior level (0.05 to 10^−3^) that would be close to what would be expected for a candidate gene; and at a very low prior level (10^−4^ to 10^−6^) that would be close to what would be expected for a random SNP.

## Additional Information

**How to cite this article**: Yu, L. *et al.* Quantitative assessment of common genetic variations in HLA-DP with hepatitis B virus infection, clearance and hepatocellular carcinoma development. *Sci. Rep.* **5**, 14933; doi: 10.1038/srep14933 (2015).

## Supplementary Material

Supplementary Information

## Figures and Tables

**Table 1:** Results of meta-analysis for rs3077 polymorphism with HBV infection outcomes.

Overall and subgroups analyses	No. of data sets	No. of cases/controls	A vs. G allele				AG vs. GG				AA vs. GG			
			OR (95% CI)	P(Z)	P(Q)	I^2^ (%)	OR (95% CI)	P(Z)	P(Q)	I^2^ (%)	OR (95% CI)	P(Z)	P(Q)	I^2^ (%)
HBV Infection (All)	28	18898/22419	0.59 (0.55–0.62)	<10^−5^	<10^−5^	67.7	0.55 (0.51–0.61)	<10^−5^	<10^−5^	62.3	0.39 (0.35–0.45)	<10^−5^	0.002	50.1
Asian	26	17918/21610	0.58 (0.55–0.62)	<10^−5^	<10^−5^	68.4	0.56 (0.51–0.61)	<10^−5^	<10^−5^	62.7	0.39 (0.35–0.44)	<10^−5^	0.005	48.5
Non-Asians	2	980/809	0.66 (0.57–0.78)	<10^−5^	0.88	0	0.39 (0.11–1.32)	0.13	0.03	79.1	0.39 (0.13–1.13)	0.08	0.05	75.0
GWAS	5	2592/5439	0.56 (0.48–0.65)	<10^−5^	0.007	71.9	0.51 (0.40–0.64)	<10^−5^	0.01	73.5	0.37 (0.26–0.53)	<10^−5^	0.03	67.4
Replication study	23	16306/16980	0.59 (0.56–0.65)	<10^−5^	<10^−5^	66.7	0.56 (0.51–0.62)	<10^−5^	<10^−5^	60.7	0.40 (0.35–0.46)	<10^−5^	0.008	48.2
Large studies	14	14356/15273	0.62 (0.57–0.67)	<10^−5^	<10^−5^	75.6	0.61 (0.55–0.67)	<10^−5^	0.003	61.3	0.44 (0.37–0.52)	<10^−5^	0.001	65.3
Small studies	14	4542/7146	0.55 (0.51–0.59)	<10^−5^	0.20	23.4	0.49 (0.43–0.56)	<10^−5^	0.05	43.0	0.34 (0.29–0.39)	<10^−5^	0.73	0
HBV Clearance (All)	22	6627/14041	1.51 (1.35–1.68)	<10^−5^	<10^−5^	76.0	1.66 (1.47–1.87)	<10^−5^	0.003	52.7	2.19 (1.80–2.67)	<10^−5^	<10^−5^	59.9
Asian	21	6327/13262	1.53 (1.37–1.71)	<10^−5^	<10^−5^	75.3	1.69 (1.50–1.90)	<10^−5^	<10^−5^	51.7	2.28 (1.87–2.77)	<10^−5^	<10^−5^	57.1
Non-Asians	1	300/779	1.12 (0.90–1.39)	0.32	NA	NA	1.03 (0.61–1.73)	0.91	NA	NA	1.17 (0.71–1.93)	0.53	NA	NA
GWAS	1	185/181	2.25 (1.62–3.13)	<10^−5^	NA	NA	2.54 (1.61–4.01)	<10^−5^	NA	NA	3.53 (1.72–7.22)	0.001	NA	NA
Replication study	21	6442/13860	1.48 (1.33–1.65)	<10^−5^	<10^−5^	75.3	1.63 (1.45–1.83)	<10^−5^	0.007	50.3	2.15 (1.76–2.62)	<10^−5^	<10^−5^	60.2
Large studies	8	4253/10412	1.51 (1.31–1.73)	<10^−5^	<10^−5^	79.9	1.66 (1.37–2.00)	<10^−5^	0.001	74.4.	2.09 (1.57–2.79)	<10^−5^	<10^−5^	75.1
Small studies	14	2374/3629	1.52 (1.27–1.82)	<10^−5^	<10^−5^	75.2	1.66 (1.42–1.94)	<10^−5^	0.17	26.7	2.31 (1.73–3.08)	<10^−5^	<10^−5^	48.3
AsC	4	1142/1388	1.04 (0.83–1.29)	0.75	0.08	56.0	1.23 (0.97–1.56)	0.09	0.30	17.5	1.25 (0.93–1.68)	0.15	0.55	0
HCC development	7	4475/6299	0.99 (0.89–1.11)	0.90	0.02	61.3	0.97 (0.83–1.14)	0.71	0.05	55.6	1.05 (0.78–1.42)	0.74	0.02	62.6

NA: not available; AsC: asymptomatic HBsAg carrier; HCC: hepatocellular carcinoma.

**Table 2:** Results of meta-analysis for rs9277535 polymorphism with HBV infection outcomes.

Overall and subgroups analyses	No. of data sets	No. of cases/controls	A vs. G allele				AG vs. GG				AA vs. GG			
			OR (95% CI)	P(Z)	P(Q)	I^2^ (%)	OR (95% CI)	P(Z)	P(Q)	I^2^ (%)	OR (95% CI)	P(Z)	P(Q)	I^2^ (%)
HBV Infection (All)	32	22065/23500	0.60 (0.57–0.63)	<10^−5^	<10^−5^	66.1	0.56 (0.52–0.60)	<10^−5^	0.01	40.7	0.39 (0.35–0.43)	<10^−5^	0.01	40.6
Asian	30	21099/22694	0.59 (0.56–0.62)	<10^−5^	<10^−5^	59.6	0.56 (0.52–0.60)	<10^−5^	0.009	43.6	0.38 (0.35–0.42)	<10^−5^	0.02	39.0
Non-Asians	2	966/806	0.85 (0.73–0.99)	0.04	0.57	0	0.62 (0.41–0.94)	0.02	0.32	0	0.59 (0.39–0.89)	0.01	0.35	0
GWAS	6	2917/5947	0.57 (0.48–0.67)	<10^−5^	<10^−5^	80.9	0.49 (0.38–0.63)	<10^−5^	0.001	77.6	0.38 (0.28–0.53)	<10^−5^	0.01	69.9
Replication study	26	19148/17553	0.60 (0.57–0.64)	<10^−5^	<10^−5^	61.7	0.57 (0.54–0.61)	<10^−5^	0.22	17.4	0.39 (0.35–0.43)	<10^−5^	0.06	32.7
Large studies	17	17211/15611	0.64 (0.59–0.68)	<10^−5^	<10^−5^	70.1	0.60 (0.57–0.64)	<10^−5^	0.29	15.0	0.43 (0.38–0.48)	<10^−5^	0.03	45.5
Small studies	15	4854/7889	0.54 (0.51–0.58)	<10^−5^	0.16	27.1	0.49 (0.44–0.54)	<10^−5^	0.24	19.3	0.32 (0.28–0.37)	<10^−5^	0.77	0
HBV Clearance (All)	25	7753/17089	1.54 (1.43–1.66)	<10^−5^	<10^−4^	62.8	1.64 (1.50–1.79)	<10^−5^	0.08	30.7	2.31 (1.99–2.67)	<10^−5^	0.003	50.4
Asian	24	7451/16324	1.57 (1.46–1.68)	<10^−5^	0.001	55.6	1.65 (1.51–1.80)	<10^−5^	0.10	29.0	2.37 (2.06–2.73)	<10^−5^	0.02	42.3
Non-Asians	1	302/765	1.02 (0.82–1.26)	0.85	NA	NA	1.08 (0.64–1.81)	0.78	NA	NA	1.07 (0.65–1.77)	0.78	NA	NA
GWAS	1	185/181	1.95 (1.42–2.69)	<10^−5^	NA	NA	2.65 (1.68–4.17)	<10^−5^	NA	NA	2.48 (1.28–4.82)	0.007	NA	NA
Replication study	24	7568/16908	1.53 (1.41–1.65)	<10^−5^	<10^−5^	62.7	1.60 (1.48–1.74)	<10^−5^	0.18	21.6	2.30 (1.98–2.69)	<10^−5^	0.002	52.5
Large studies	10	4443/12359	1.47 (1.36–1.60)	<10^−5^	0.04	50.1	1.52 (1.38–1.68)	<10^−5^	0.22	25.3	2.12 (1.78–2.54)	<10^−5^	0.04	51.4
Small studies	15	3310/4730	1.61 (1.40–1.85)	<10^−5^	<10^−5^	67.1	1.82 (1.59–2.08)	<10^−5^	0.36	8.7	2.52 (1.99–3.17)	<10^−5^	0.04	44.7
AsC	6	1751/2706	0.98 (0.87–1.09)	0.66	0.33	13.9	0.97 (0.84–1.12)	0.65	0.93	0	1.04 (0.71–1.52)	0.85	0.06	53.1
HCC development	8	4398/7358	1.03 (0.97–1.09)	0.35	0.59	0	0.92 (0.84–1.02)	0.10	0.93	0	1.11 (0.95–1.29)	0.19	0.66	0

NA: not available; AsC: asymptomatic HBsAg carrier; HCC: hepatocellular carcinoma.
