# Supplementary material for: Quantitative assessment of common genetic variations in HLA-DP with hepatitis B virus infection, clearance and hepatocellular carcinoma development
Source: Sci Rep. 2015 Oct 14;5:14933. doi: 10.1038/srep14933 (PMC4604517; doi:10.1038/srep14933)
Supplement: Supplementary Information [file srep14933-s1.docx]

**Quantitative assessment of common genetic variations in HLA-DP with hepatitis B virus infection, clearance and hepatocellular carcinoma development**

Lei Yu, Yi-ju Cheng, Ming-liang Cheng^*^, Yu-mei Yao, Quan Zhang, Xue-ke Zhao, Hua-juan Liu, Ya-xin Hu, Mao Mu, Bi Wang, Guo-zhen Yang^*^, Li-li Zhu, Shuai Zhang

*Corresponding author

**Supplementary table 1** Characteristics of the studies included in the meta-analysis

| Study | Year | Ethnic population | Subgroup | Cases | Controls | No. of cases/controls | Mean age of cases/controls | Sex distribution in cases/control (male %) | Genotyping method | Quality score |
| --- | --- | --- | --- | --- | --- | --- | --- | --- | --- | --- |
| Kamatani | 2009 | Japanese | GWAS second stage | CHB | HBsAg (-) | 606/1267 | 59.6/46.7 | 61.4/36.2 | GeneChip | 9 |
|  |  |  | First replication | CHB | HBsAg (-) | 272/272 | 55.3/55.3 | 66.8/66.8 | Invader assay |  |
|  |  |  | Second replication | CHB | HBsAg (-) | 711/1278 | 46.6/60.9 | 68.0/53.2 | TaqMan |  |
|  |  |  | Third replication | CHB | HBsAg (-) | 304/545 | 57.0/57.1 | 84.4/83.5 | TaqMan |  |
| An | 2011 | Chinese |  | HBV carriers (CHB, LC, HCC) | HBV natural clearances, Healthy individuals | 1218/287, 227 | 48.3/50.0, 48.7 | 62.2/70.0, 61.7 | TaqMan | 7 |
| Wang | 2011 | Chinese | Han | CHB | HBV natural clearances | 736/782 | 35.5/43.2 | 66.9/65.0 | TaqMan | 7 |
|  |  |  | Zhuang | CHB | HBV natural clearances | 177/208 | 41.2/39.4 | 57.1/64.4 | TaqMan |  |
| Lau | 2011 | Chinese |  | CHB | non-CHB | 216/231 | NR/NR | 87.5/NR | HRM | 6 |
| Li | 2011 | Chinese | South | HBV carriers (CHB, AsC, LC, HCC) | HBV natural clearances, Healthy individuals | 2202/526, 573 | NR/NR | NR/NR | TaqMan | 8 |
|  |  |  | North | HBV carriers (CHB, AsC, LC, HCC ) | HBV natural clearances, Healthy individuals | 611/307, 380 | NR/NR | NR/NR | TaqMan |  |
| Guo | 2011 | Chinese |  | HBV carriers | HBV natural clearances, Healthy individuals | 514/562, 808 | 41.0/50.0, 47.0 | 51.4/42.6, 37.1 | TaqMan | 8 |
| Mbarek | 2011 | Japanese | GWAS | CHB | non-HBV | 458/2056 | 56.6/51.9 | 69.4/44.3 | GeneChip | 8 |
|  |  |  | First replication | CHB | non-HBV | 606/2023 | 55.6/60.5 | 61.4/77.0 | TaqMan, Invader assay |  |
|  |  |  | Second replication | CHB | non-HBV | 379/1539 | 54.4/67.6 | 64.9/66.7 | TaqMan, Invader assay |  |
|  |  |  | Third replication | CHB | non-HBV | 1226/879 | 47.9/40.2 | 66.4/34.2 | TaqMan, Invader assay |  |
| Migita | 2012 | Japanese |  | HBV carriers | Healthy individuals | 241/143 | NR/31.3 | NR/39.2 | TaqMan | 6 |
| Hu | 2012 | Chinese |  | HBV-positive HCC, HBV carriers | HBV natural clearances | 2627/1328 | 52.8/52.6 | 84.8/83.8 | TaqMan | 8 |
| Vermehren | 2012 | German |  | HBV carriers ( AsC) | Healthy individuals | 201/235 | 42.0/NR | 63.7/NR | Sequencing | 7 |
| Nishida | 2012 | Japanese, Korean | GWAS | HBV carriers | HBV natural clearances, Healthy individuals | 181/185, 183 | NR/NR | NR/NR | GeneChip | 8 |
|  |  |  | Japanese | HBV carriers | HBV natural clearances, Healthy individuals | 255/144, 236 | NR/NR | NR/NR | DigiTag2 assay, TaqMan |  |
|  |  |  | Korean | HBV carriers | HBV natural clearances, Healthy individuals | 215/105, 145 | NR/NR | NR/NR | DigiTag2 assay, TaqMan |  |
| Yan | 2012 | Chinese |  | CHB | Spontaneously recovered carriers | 280/64 | 41.4/39.2 | 74.4/57.8 | TaqMan | 6 |
| Jiang | 2013 | Chinese |  | HCC | HBV carriers | 1161/1353 | 51.6/48.4 | 77.3/67.8 | GeneChip | 9 |
| Seto | 2013 | Chinese |  | HBV carriers | HBV natural clearances | 203/203 | 52.2/51.9 | 70.4/70.4 | TaqMan | 6 |
| Zhang | 2013 | Chinese |  | HBV carriers | HBsAg seroclearance, Healthy individuals | 2691/326, 1330 | 52.0/57.5, 53.1 | 72.0/56.9, 71.0 | HRM | 8 |
| Cheng | 2013 | Chinese |  | HBV carriers | HBV natural clearances | 100/100 | 53.3/53.8 | 100/100 | TaqMan | 6 |
| Hu | 2013 | Chinese | GWAS | HBV carriers | HBsAg (-) | 951/937 | 49.6/50.8 | 83.2/56.2 | GeneChip | 9 |
|  |  |  | Replication Ia | HBV carriers | HBsAg (-) | 1245/1236 | 49.6/49.6 | 51.3/51.3 | iPLEX |  |
|  |  |  | Replication Ib | HBV carriers | HBsAg (-) | 997/1803 | 52.2/52.5 | 49.3/49.2 | TaqMan |  |
| Wong | 2013 | Chinese |  | HBV carriers | HBV natural clearances, Healthy individuals | 500/259, 245 | 46.8/40.3, 36.4 | 61.0/59.0, 52.0 | TaqMan | 7 |
| Kim | 2013 | Korean | GWAS | CHB | Healthy individuals | 400/1000 | 55.1/52.2 | 78.0/78.3 | GeneChip | 8 |
|  |  |  | Replication | CHB | Healthy individuals | 971/1938 | 47.4/52.2 | 62.5/47.2 | TaqMan |  |
| Al-Qahtani | 2014 | Saudi Arabian |  | HBV carriers (AsC, LC, HCC) | HBV natural clearances, Healthy individuals | 779/302, 587 | 41.0/37.0, 29.0 | 72.7/99.0, 94.4 | TaqMan | 7 |
| Posuwan | 2014 | Thai |  | HBV carriers | HBV natural clearances, Healthy individuals | 449/113, 123 | 52.5/48.2, 46.7 | 74.4/73.5, 59.3 | TaqMan | 7 |
| Liao | 2014 | Chinese |  | HBV-positive HCC, HBV carriers | HBV natural clearances, Healthy individuals | 661/396, 237 | 47.0/49.0, 42.0 | 70.6/51.2, 38.0 | HRM | 7 |
| Hosaka | 2015 | Japanese | cohort 1 | HBV carriers | HBsAg seroclearance | 87/11 | 41.0/45.0 | 69.0/81.8 | TaqMan | 6 |
|  |  |  | cohort 2 | HBV carriers | HBsAg seroclearance | 97/7 | 43.0/44.0 | 77.3/100 | TaqMan |  |
| Chang | 2014 | Chinese | GWAS | HBV carriers | Healthy individuals | 321/304 | 51.0/48.8 | 100/100 | GeneChip | 8 |
|  |  |  | 1st replication | HBV carriers | Healthy individuals | 646/345 | 49.6/52.3 | 100/100 | iPLEX, TaqMan |  |
|  |  |  | 2nd replication | HBV carriers | Healthy individuals | 656/416 | 50.6/55.0 | 100/100 | iPLEX, TaqMan |  |
| He | 2014 | Chinese |  | HBV carriers | HBV natural clearances | 1186/272 | 36.0/42.0 | 64.4/59.3 | iMLDR | 7 |
| Hu | 2014 | Chinese |  | HBV carriers ( AsC, LC, CHB) | HBV natural clearances | 342/342 | 41.4/41.6 | 48.2/48.2 | SNaPshot | 6 |
| Su | 2014 | Chinese |  | HBV carriers ( AsC, LC, CHB) | HBV natural clearances, Healthy individuals | 476/255, 204 | 41.8/46.5, 45.4 | 65.7/52.2, 47.1 | PCR-SSP | 6 |
| Liao | 2015 | Chinese | Tibetans | HBV carriers | HBV natural clearances | 420/485 | 39.0/39.0 | 68.4/63.6 | HRM | 7 |
|  |  |  | Uygurs | HBV carriers | HBV natural clearances | 192/235 | 29.0/48.0 | 61.6/60.0 | HRM |  |
| Unpublished data ^a^ |  | Chinese |  | HBV carriers ( AsC, LC, HCC) | Healthy individuals | 1184/929 | 39.3/52.3 | 73.1/49.8 | PCR-RFLP | 7 |

^a^ Identified from PhD dissertations with unpublished data

CHB: Chronic Hepatitis B; HBV: Hepatitis B Virus; HCC: Hepatocellular Carcinoma; LC: Liver Cirrhosis; AsC: Asymptomatic Carriers; NR: Not Reported

**Supplementary table 2** Haplotype analysis for HLA-DP gene polymorphisms and chronic HBV infection, with GG as the reference

| Study | Ethnicity | Subjects | | G-G | | A-A | | | | A-G | | | | G-A | | | |
| --- | --- | --- | --- | --- | --- | --- | --- | --- | --- | --- | --- | --- | --- | --- | --- | --- | --- |
|  |  |  |  | Frequency | | Frequency | |  | | Frequency | |  | | Frequency | |  | |
|  |  | Case | Control | Case | Control | Case | Control | OR (95% CI) | P | Case | Control | OR (95% CI) | P | Case | Control | OR (95% CI) | P |
| Kamatani (GWAS second stage) | Japanese | 576 | 867 | 0.701 | 0.577 | 0.241 | 0.351 | 0.57 (0.45-0.72) |  | 0.012 | 0.017 | 0.58(0.23-1.43) |  | 0.045 | 0.055 | 0.67 (0.41-1.10) |  |
| Guo | Chinese | 371 | 611 | 0.551 | 0.394 | 0.292 | 0.434 | 0.48 (0.39-0.59) |  | 0.022 | 0.017 | 0.97(0.50-1.86) |  | 0.135 | 0.155 | 0.62 (0.47-0.82) |  |
| Wong | Chinese | 490 | 240 | 0.714 | 0.619 | 0.150 | 0.221 | 0.59 (0.44-0.78) |  | 0.054 | 0.058 | 0.80(0.50-1.30) |  | 0.082 | 0.102 | 0.69 (0.47-1.01) |  |
| Al-Qahtani | Saudi Arabian | 781 | 587 | 0.162 | 0.128 | 0.624 | 0.657 | 0.75 (0.55-1.03) |  | 0.108 | 0.110 | 0.78(0.51-1.20) |  | 0.105 | 0.105 | 0.78 (0.50-1.20) |  |
| Liao | Chinese | 403 | 221 | 0.661 | 0.481 | 0.187 | 0.250 | 0.58 (0.44-0.78) |  | 0.085 | 0.080 | 0.84(0.55-1.29) |  | 0.080 | 0.139 | 0.45 (0.31-0.66) |  |
| Su | Chinese | 476 | 204 | 0.662 | 0.542 | 0.210 | 0.297 | 0.58(0.44-0.76) |  | 0.050 | 0.051 | 0.86 (0.49-1.53) |  | 0.078 | 0.110 | 0.60 (0.40-0.90) |  |
| Pooled from random effects model | All | 3097 | 2730 | / | / | / | / | 0.57 (0.51-0.64) | < 10^-5^ | / | / | 0.82(0.66-1.01) | 0.06 | / | / | 0.62 (0.53-0.72) | < 10^-5^ |

SNP order of haplotype: rs3077, rs9277535.

**Supplementary table 3** Haplotype analysis for HLA-DP gene polymorphisms and clearance of HBV, with GG as the reference

| Study | Ethnicity | Subjects | | G-G | | A-A | | | | A-G | | | | G-A | | | |
| --- | --- | --- | --- | --- | --- | --- | --- | --- | --- | --- | --- | --- | --- | --- | --- | --- | --- |
|  |  |  |  | Frequency | | Frequency | |  | | Frequency | |  | | Frequency | |  | |
|  |  | Case | Control | Case | Control | Case | Control | OR (95% CI) | P | Case | Control | OR (95% CI) | P | Case | Control | OR (95% CI) | P |
| Hu | Chinese | 1171 | 2328 | 0.558 | 0.653 | 0.321 | 0.240 | 1.56 (1.40-1.75) |  | NA | NA | NA |  | 0.122 | 0.106 | 1.34 (1.14-1.57) |  |
| Liao (Tibetan) | Chinese | 486 | 413 | 0.427 | 0.478 | 0.463 | 0.369 | 1.40 (1.07-1.83) |  | 0.036 | 0.047 | 0.85 (0.45-1.60) |  | 0.040 | 0.046 | 0.98 (0.52-1.83) |  |
| Liao (Uygur) | Chinese | 171 | 179 | 0.157 | 0.245 | 0.648 | 0.506 | 2.01 (1.16-3.49) |  | 0.080 | 0.103 | 1.23 (0.53-2.84) |  | 0.115 | 0.128 | 1.45 (0.67-3.12) |  |
| Wang (Han) | Chinese | 782 | 736 | 0.435 | 0.576 | 0.309 | 0.216 | 1.89 (1.59-2.25) |  | 0.120 | 0.065 | 2.42 (1.85-3.15) |  | 0.138 | 0.143 | 1.28 (1.03-1.58) |  |
| Wang (Zhuang) | Chinese | 208 | 177 | 0.577 | 0.664 | 0.151 | 0.096 | 1.94 (1.24-3.03) |  | 0.084 | 0.102 | 0.77 (0.46-1.30) |  | 0.188 | 0.138 | 1.40 (0.94-2.10) |  |
| Cheng | Chinese | 100 | 100 | 0.695 | 0.582 | 0.160 | 0.196 | 1.46 (0.86-2.48) |  | 0.055 | 0.058 | 1.31 (0.56-3.07) |  | 0.090 | 0.164 | 2.20 (1.18-4.10) |  |
| Wong | Chinese | 255 | 490 | 0.600 | 0.714 | 0.212 | 0.150 | 1.68 (1.27-2.23) |  | 0.059 | 0.054 | 1.29 (0.81-2.06) |  | 0.219 | 0.082 | 1.88 (1.32-2.68) |  |
| Yan | Chinese | 64 | 276 | 0.453 | 0.630 | 0.305 | 0.178 | 2.39 (1.50-3.80) |  | 0.102 | 0.089 | 1.59 (0.81-3.12) |  | 0.141 | 0.103 | 1.89 (1.04-3.45) |  |
| Liao | Chinese | 331 | 403 | 0.511 | 0.640 | 0.178 | 0.158 | 1.42 (1.07-1.87) |  | 0.096 | 0.085 | 1.41 (0.99-2.01) |  | 0.131 | 0.080 | 2.05 (1.46-2.88) |  |
| Su | Chinese | 476 | 255 | 0.662 | 0.541 | 0.210 | 0.288 | 1.68 (1.30-2.16) |  | 0.050 | 0.051 | 1.19 (0.70-2.01) |  | 0.078 | 0.117 | 1.86 (1.28-2.70) |  |
| Pooled from random effects model | All | 4044 | 5357 | / | / | / | / | 1.65 (1.52-1.79) | < 10^-5^ | / | / | 1.32 (0.98-1.76) | 0.06 | / | / | 1.54 (1.34-1.78) | < 10^-5^ |

SNP order of haplotype: rs3077, rs9277535.

NA: not available

**Supplementary table 4** Bayesian false discovery probability for rs3077 and HBV infection and spontaneous viral clearance.

| ***OR (95% CI)*** | ***Observed p-value*** | ***Prior probabilities*** | | | | | |
| --- | --- | --- | --- | --- | --- | --- | --- |
| rs3077 |  | 0.05 | 0.01 | 10^-3^ | 10^-4^ | 10^-5^ | 10^-6^ |
| HBV Infection |  | **Bayesian False Discovery Probability** | | | | | |
| 0.59 (0.55-0.62) | P < 10^-5^ | < 10^-3^ | < 10^-3^ | < 10^-3^ | < 10^-3^ | < 10^-3^ | < 10^-3^ |
| HBV Clearance |  | **Bayesian False Discovery Probability** | | | | | |
| 1.51 (1.35-1.68) | P < 10^-5^ | < 10^-3^ | < 10^-3^ | < 10^-3^ | < 10^-3^ | < 10^-3^ | < 10^-3^ |

**Supplementary table 5** Bayesian false discovery probability for rs9277535 and HBV infection and spontaneous viral clearance.

| ***OR (95% CI)*** | ***Observed p-value*** | ***Prior probabilities*** | | | | | |
| --- | --- | --- | --- | --- | --- | --- | --- |
| rs9277535 |  | 0.05 | 0.01 | 10^-3^ | 10^-4^ | 10^-5^ | 10^-6^ |
| HBV Infection |  | **Bayesian False Discovery Probability** | | | | | |
| 0.60 (0.57-0.63) | P < 10^-5^ | < 10^-3^ | < 10^-3^ | < 10^-3^ | < 10^-3^ | < 10^-3^ | < 10^-3^ |
| HBV Clearance |  | **Bayesian False Discovery Probability** | | | | | |
| 1.54 (1.43-1.66) | P < 10^-5^ | < 10^-3^ | < 10^-3^ | < 10^-3^ | < 10^-3^ | < 10^-3^ | < 10^-3^ |

**Supplementary table 6** Results of Venice criteria grade for rs3077 and rs9277535

| Group | Venice criteria grade |
| --- | --- |
| rs3077 |  |
| HBV Infection | AAA |
| HBV Clearance | AAA |
| rs9277535 |  |
| HBV Infection | AAA |
| HBV Clearance | AAA |

**Supplementary Figure 1**


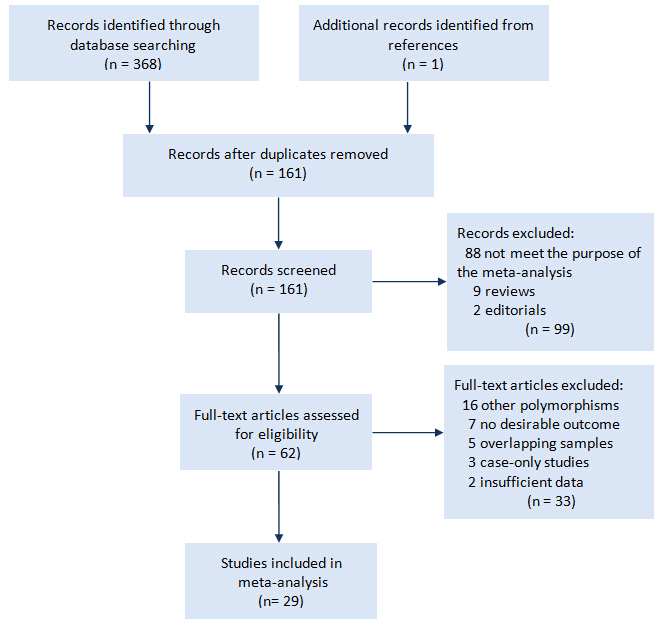


**Supplementary figure 1** Flow chart of literature search for studies examining polymorphisms at HLA-DP and outcomes of HBV infection.

**Supplementary figure 2**

**
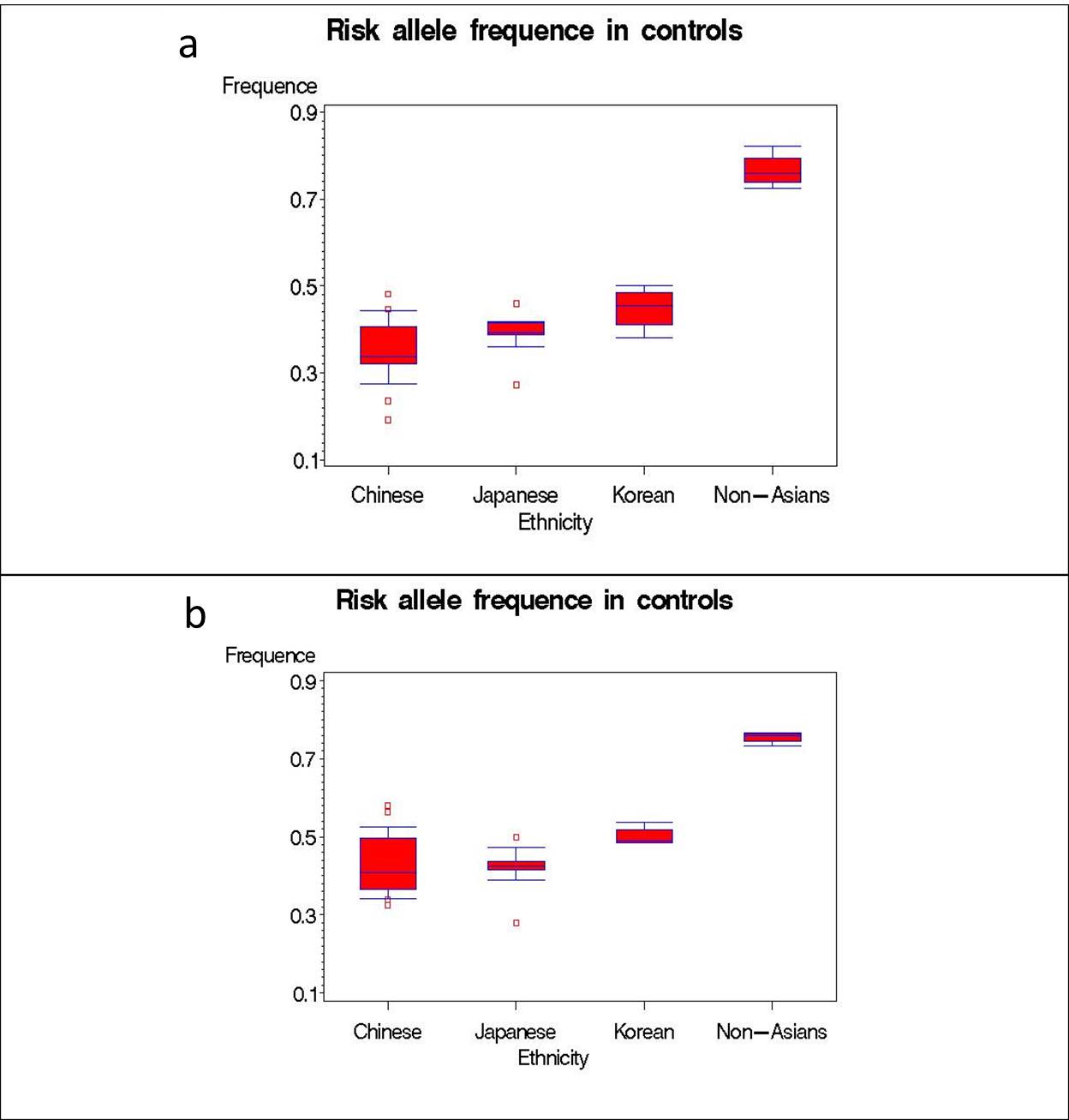
**

**Supplementary figure 2** Frequencies of the risk allele of rs3077 and rs9277535 polymorphism among controls stratified by ethnicity. The ‘‘□’’ represent outlier.

(a) A-allele frequency of rs3077; (b) A-allele frequency of rs9277535.

**Supplementary figure 3**

**Supplementary figure 3** Galbraith plots for heterogeneity test of rs3077 polymorphism and HBV infection.

**Supplementary figure 4**

**Supplementary figure 4** Galbraith plots for heterogeneity test of rs3077 polymorphism and HBV clearance.

**Supplementary figure 5**

**Supplementary figure 5** Galbraith plots for heterogeneity test of rs9277535 polymorphism and HBV infection.

**Supplementary figure 6**

**Supplementary figure 6** Galbraith plots for heterogeneity test of rs9277535 polymorphism and HBV clearance.

**Supplementary figure 7**


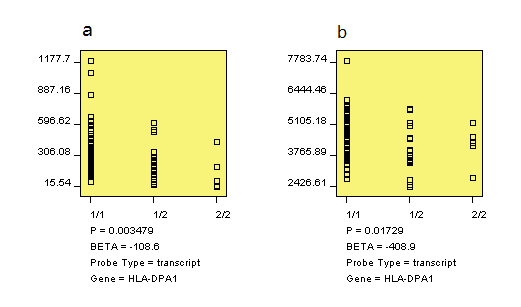


**Supplementary figure 7** The mRNA expression level of HLA-DPA1 by the genotypes of rs3077 polymorphism

(a) HLA-DPA1 mRNA expression level in peripheral blood mononuclear cells (PBMC) stratified by genotypes of rs3077; (b) HLA-DPA1 mRNA expression level in brain tissues stratified by genotypes of rs3077 (1 = A allele, 2 = G allele).

**Supplementary figure 8**

**Supplementary figure 8** Result of sensitivity analyses for rs3077 polymorphism and HBV infection risk (A vs. G allele).

**Supplementary figure 9**

**Supplementary figure 9** Result of sensitivity analyses for rs3077 polymorphism and HBV clearance risk (A vs. G allele).

**Supplementary figure 10**

**Supplementary figure 10** Result of sensitivity analyses for rs9277535 polymorphism and HBV infection risk (A vs. G allele).

**Supplementary figure 11**

**Supplementary figure 11** Result of sensitivity analyses for rs9277535 polymorphism and HBV clearance risk (A vs. G allele).

**Supplementary figure 12**

**Supplementary figure 12** Funnel plot of studies of the rs3077 polymorphism and HBV infection (A vs. G allele); Egger’s test was also performed to investigate the symmetry of the funnel plot (P = 0.18).

**Supplementary figure 13**

**Supplementary figure 13** Funnel plot of studies of the rs3077 polymorphism and HBV clearance (A vs. G allele); Egger’s test was also performed to investigate the symmetry of the funnel plot (P = 0.38).

**Supplementary figure 14**

**Supplementary figure 14** Funnel plot of studies of the rs9277535 polymorphism and HBV infection (A vs. G allele); Egger’s test was also performed to investigate the symmetry of the funnel plot (P = 0.26).

**Supplementary figure 15**

**Supplementary figure 15** Funnel plot of studies of the rs9277535 polymorphism and HBV clearance (A vs. G allele); Egger’s test was also performed to investigate the symmetry of the funnel plot (P = 0.06).
